# Association between Repeated Unpredictable Chronic Mild Stress (UCMS) Procedures with a High Fat Diet: A Model of Fluoxetine Resistance in Mice

**DOI:** 10.1371/journal.pone.0010404

**Published:** 2010-04-28

**Authors:** Elsa Isingrini, Vincent Camus, Anne-Marie Le Guisquet, Maryse Pingaud, Séverine Devers, Catherine Belzung

**Affiliations:** 1 UMRS (Unité mixte de recherche) INSERM U930 (Institut national de la santé et de la recherche médicale), CNRS (Centre national de la recherche scientifique) ERL (Equipes de recherche labélisées) 3106, Université François Rabelais, Tours, France; 2 Clinique Psychiatrique Universitaire, CHRU (Centre hospitalier régional universitaire) de Tours, Tours, France; L'université Pierre et Marie Curie, France

## Abstract

Major depressive disorder is a debilitating disease. Unfortunately, treatment with antidepressants (ADs) has limited therapeutic efficacy since resistance to AD is common. Research in this field is hampered by the lack of a reliable natural animal model of AD resistance. Depression resistance is related to various factors, including the attendance of cardiovascular risk factors and past depressive episodes. We aimed to design a rodent model of depression resistance to ADs, associating cardiovascular risk factors with repeated unpredicted chronic mild stress (UCMS). Male BALB/c mice were given either a regular (4% fat) or a high fat diet (45% fat) and subjected to two 7-week periods of UCMS separated by 6 weeks. From the second week of each UCMS procedure, vehicle or fluoxetine (10 mg/kg, i.p.) was administrated daily. The effects of the UCMS and fluoxetine in both diet conditions were assessed using physical (coat state and body weight) and behavioural tests (the reward maze test and the splash test). The results demonstrate that during the second procedure, UCMS induced behavioural changes, including coat state degradation, disturbances in self-care behaviour (splash test) and anhedonia (reward maze test) and these were reversed by fluoxetine in the regular diet condition. In contrast, the high-fat diet regimen prevented the AD fluoxetine from abolishing the UCMS-induced changes. In conclusion, by associating UCMS—an already validated animal model of depression—with high-fat diet regimen, we designed a naturalistic animal model of AD resistance related to a sub-nosographic clinical entity of depression.

## Introduction

Major depressive disorder is a debilitating disease with a prevalence estimated to be as high as 16.2% according to the National Comorbidity Survey [1]. Unfortunately, the therapeutic efficacy of antidepressants (ADs) is unsatisfactory, since most patients fail to achieve a full remission when treated, mostly being non- or partial responders. Remission (i.e., full resolution of symptoms) occurs in only one third of the patients after treatment with a single drug [2], [3]. AD resistance is related to various factors, including specific diagnostic entities. AD resistance is more frequent in some sub-nosographic disorders such as vascular depression. In this subtype of depression, it is suggested that the presence of brain cortical lesion of vascular origin predisposes, precipitates, or perpetuates a depressive state and has a negative impact on treatment outcome [4], [5], [6].

Furthermore, treatment-resistant major depression is also frequently described in patients with acute coronary heart disease, and seems to increase risk of mortality after acute coronary syndrome [7]. Moreover, cardiovascular risk factors like aging, smoking, hypertension, diabetes, hypercholesterolemia and advanced heart disease predict a poor response to fluoxetine/citalopram treatment and lack of remission [8], [9]. Although low cholesterol levels have been reported in depressed subjects [10], [11], in several studies, hypercholesterolemia, a risk factor for cardiovascular disease, was associated with a poor outcome following AD in major depression [12], [13]. Finally, it seems that an increased number of past depressive episodes can also be associated with resistance to AD [14], [15]. According to these results it seems that both the occurrence of vascular risk factors and the number of repeated depressive episodes can increase the risk of resistance to AD in major depression. However, research in this field is hampered by the lack of animal models of AD-resistance.

Several animal models of treatment-resistant depression have been proposed. Some are based on the invalidation of genes encoding for proteins involved either in the brain ADs penetration [16] or in the ADs molecular target [17], [18], [19], [20]. In some instances, mutant mice only show a blunted response to AD and not a total suppression of the AD effect [21], [22], [23], [24]. The main limit of this approach, based on the invalidation of single gene, is that it only partially mimics the human condition. Other approaches looked at the alteration of particular behavioural markers (for example high responders to novelty decreased response to desipramine in rats: [25] or bio-markers (mice with a disrupted neurogenesis do not respond to monoaminergic AD: [17], [26]. However, all of these models are mostly concerned with providing a mechanism explaining the AD resistance and not in proposing a naturalistic animal model of AD resistance related to sub-nosographic clinical entities.

For studying depressive disorders, the unpredictable chronic mild stress (UCMS) animal model has been shown to be valid, reliable and sensitive [27]. UCMS involves subjecting mice to a period of mild socio-environmental stressors. This procedure replicates several depression-related behavioural and physiological impairments which are reversed by chronic (but not acute) AD treatment [28], [29], [30], [31]. Furthermore, since cardiovascular risk factors are related to AD resistance, the association of the UCMS procedure with a cardiovascular risk factor known to induce hypercholesterolemia in mice such as a high fat diet regimen provides a powerful tool to describe a model of “treatment-resistant depression”. The development of a naturalistic animal model of AD resistance related to sub-nosographic clinical entities will contribute to the development of new drugs which may be useful in the treatment of vascular depression.

The main objective of the present study was to evaluate the ability of chronic AD treatment with fluoxetine to reverse UCMS-induced depression-like behaviour in BALB/c mice when associating both repeated episodes of UCMS and a high fat diet regimen. As a 6-month period of a high fat diet regimen is necessary to induce cardiovascular alterations in BALB/c mice [32], we planned to repeat the UCMS procedure twice in this time period, with a UCMS-free period between the two. We evaluated the response to both UCMS and high fat diet via several validated measures, including physical measures (coat state degradation and body weight) and behavioural tests such as the splash test (decease in total grooming time) and the reward maze test (decreased latency to chew a chocolate cookie).

## Methods

### Animals

The subjects were 71 experimentally naive male BALB/c mice (7–9 weeks old) (Centre d'élevage Janvier, Le Genest Saint Isle, France) housed in groups of 4 and maintained under standard laboratory conditions (12 hrs. light-dark cycle, light on at 20:00; Temperature  = 22 +/−2°C) for 1 week prior to the beginning of the experiment. Food and water were freely available. All behavioural testing occurred during the dark phase of the light–dark cycle. All of the experiments are in agreement with the veterinary service (agreement number: B37-261-2) and were carried out in strict compliance with the European Community Council directive 86/609/EEC and with French legislation from the Ministère de l'Agriculture.

### Drug

The selective serotonin reuptake inhibitor Fluoxetine hydrochloride (Sequoia) was freshly prepared every day in saline (NaCl 0.9%) and administrated intraperitoneally (IP, 10 mg/kg) in a volume of 10 ml/kg. Vehicle animals received an IP injection of 0.9% saline in a volume of 10 ml/kg.

### General procedure (Figure 1)

The mice were randomized into regular diet (RD, 4% fat, DIETEX France) and high fat diet (HFD, 45% fat and 0.15% cholesterol, DIETEX France) conditions. In each condition, the mice were divided in four groups: Control/NaCl (RD: n = 9 and HFD: n = 10), Control/Fluoxetine (RD: n = 10, HFD: n = 8), UCMS/NaCl (RD: n = 8, HFD: n = 10), and UCMS/Fluoxetine (RD: n = 6, HFD: n = 10). The control group was maintained in standard laboratory conditions while the mice in the UCMS group were placed in small, individual cages (8×13.5×8.1 cm). We performed two 7-week UCMS procedures separated by a 6 week stress- and drug-free period in the UCMS groups. The first two weeks of the UCMS regimen were drug-free. Treatment was initiated on the third week of UCMS and continued up to the end of the 7 weeks. The same procedure was repeated during the second UCMS regimen. Coat state and body weight was assessed weekly until the end of the second UCMS regimen. During the two UCMS procedures, behavioural tests were carried out as follows: the splash test and the reward maze test.

**Figure 1:**
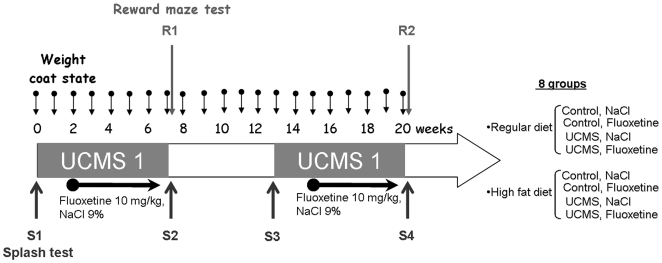
General procedure and experimental groups. In each regular diet and high fat diet condition, half of the mice were submitted to two 7-week periods of unpredictable chronic mild stress (UCMS) procedures separated by 6 weeks. Control mice were kept in standard laboratory conditions. After two weeks of UCMS, mice received either fluoxetine treatment (10 mg/kg, i.p.) or vehicle treatment (NaCl, 0.9%, i.p.). Treatments were administered daily during the last 5 weeks of the UCMS protocol and the same treatment procedure was performed during the second UCMS phase. Coat state and body weight were evaluated weekly and behaviour was assessed using the reward maze test and the splash test. One session of the reward maze test was performed at the end of the first UCMS procedure (Session R1) and one session at the end of the second UCMS procedure (Session R2). The splash test was performed at the beginning (session S1 for the first UCMS procedure and session S3 for the second UCMS procedure) and the end (session S2 for the first UCMS procedure and session S4 for the second UCMS procedure) of each UCMS procedure.

### Unpredictable chronic mild stress

The mice were subjected to various and repeated unpredictable stressors several times a day during the two 7-week UCMS procedures. The stressors were: altered bedding (change or removal of sawdust, damp sawdust, substitution of sawdust with 21°C water), cage tilting (45°), cage exchange (mice were placed in the empty cage of another male), altered length and time of light/dark cycle [26], [31], [33].

### Coat state and body weight

The coat state and the body weight of each animal were evaluated weekly until the end of the second UCMS procedure. The coat-state evaluation involved the assessment of eight different body parts: head, neck, dorsal coat, ventral coat, tail, forepaws, hind paws and genital region. For each body area, a score of 0 was attributed for a coat in good condition or a score of 1 for a dirty coat. The total score was defined as the sum of the scores for each body part. This index has been pharmacologically validated in previous studies using BALB/c mice [26], [33], [34].

### Splash Test

The splash test, performed under a red light (230 V, 15 W), consists of squirting a 10% sucrose solution on the dorsal coat of a mouse in its home cage. Because of its viscosity, the sucrose solution dirties the mouse fur and animals initiate grooming behaviour. After applying sucrose solution, the time spent grooming was recorded for a period of 5 minutes as an index of self-care and motivational behaviour. The splash test was carried out at the beginning (S1 for UCMS 1 and S3 for UCMS 2) and at the end (S2 for UCMS1 and S4 for UCMS 2) of each UCMS procedure. The splash test, pharmacologically validated, demonstrates that UCMS decreases grooming behaviour [17], [26], [31], [35], a form of motivational behaviour considered to parallel with some symptoms of depression such as apathetic behaviour [36]. Moreover, UCMS-induced grooming perturbation is associated with hedonic reactivity in the sucrose preference test and increased immobility in the force swim test [37], [38].

### Reward maze test

The reward maze test is used to assess UCMS-induced effects on the motivation to obtain a reward. This test consists in assessing the motivation for a palatable stimulus (a chocolate cookie) by measuring the latency before chewing the cookie. The reward maze test requires a device containing three aligned chambers with the same dimensions (19×19×20 cm). The only difference in the chambers is the colour of the walls and the floor: white for the first chamber, grey for the second and black for the third. The three chambers are linked by two openings via a door which is controlled by the experimenters. The device is illuminated by a 200-lux white light. To familiarize the mice with the palatable stimulus, small portion of cookie is placed in the homecage every 2 day for a period of 2.5 weeks starting 4.5 weeks before the first session. At the time of testing, a small piece of chocolate cookie is positioned at the centre of the black room. The white room is the departure compartment and the mouse is placed with its head facing away from the opening. The test lasts five minutes maximum; the door separating the departure chamber and the intermediate chamber was closed after the transition of the mouse (if a mouse did not enter after 2 minutes, it was gently guided toward the intermediate room).

The validation of this test was demonstrated by the stronger drive to chew a chocolate cookie than to chew a regular food pellet as a robust reduction of the chewing latency with the chocolate cookie when compared with the regular food. In the UCMS paradigm, the reward maze test can assess multiple behavioural dimensions: 1) anxiety-like state (latency to pass the first door), 2) locomotion and exploratory behaviour (number of passage trough the second door) and 3) anhedonia (latency to chew the chocolate cookie in the UCMS mice *vs* Control mice). This test was validated in previous study in our laboratory demonstrating that a 7-week UCMS has no effect on the anxiety-like and exploratory behaviour but significantly increases the latency to chew the cookie. These results indicate that UCMS induces anhedonia [31].

In our experiment, we performed one session at the end of each UCMS procedure (R1 at the end of the first UCMS procedure and R2 at the end of the second UCMS procedure). During the two sessions, we recorded: 1) the latency to pass the first door as an index of anxiety behaviour, 2) the number of passage through the second door as an index of locomotion and exploratory behaviour and 3) the latency to chew the cookie as an index of anhedonia.

### Statistical analysis

The results are expressed as the mean +/− SEM (standard error of the mean). Since the sample-size was small (6<n<10) and did not follow a normal distribution, we used a non-parametric analysis. Behaviour among all groups was compared using the Kruskall-Wallis test and compared among the different sessions in each group using the Friedman test (multiple samples). The Kruskall-Wallis was followed by the U of Mann-Witney test and the Friedman test was followed by the Wilcoxon test for two per two comparisons when required. Differences were considered as statistically significant at p<.05.

## Results

### Coat state and body weight

**Figure 2:**
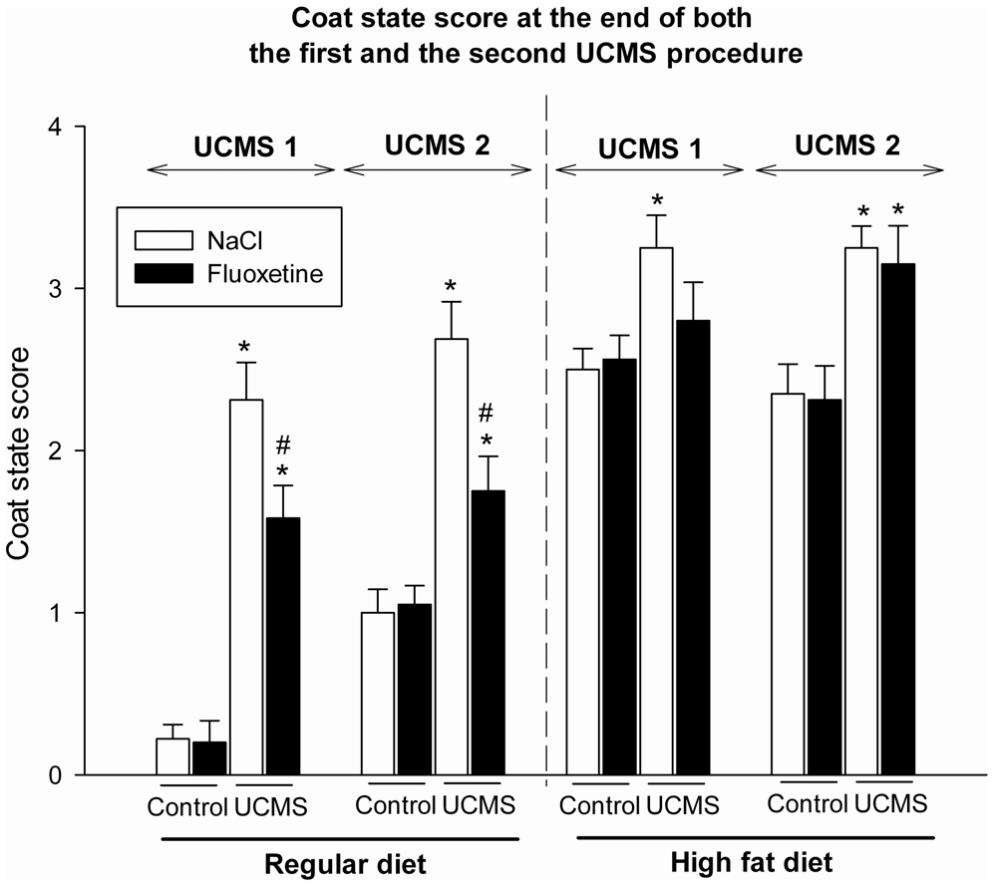
Coat state changes. The coat state scores (Mean +/− SEM) at the end of the two unpredictable chronic mild stress (UCMS) procedures are presented for both the regular diet (left) and the high fat diet (right) conditions for Control and UCMS groups treated or not with fluoxetine (10 mg/kg, administrated daily during the last 5 weeks of each UCMS procedure). *p<.05: comparison between the control and UCMS groups, from the same diet and treatment conditions. #p<.05: comparison between NaCl and fluoxetine-treated groups, from the same UCMS condition.

The statistical analysis of changes in coat state (Figure 2) between the regular diet and the high fat diet groups were performed separately because of the texture difference between the two diets. The high fat diet was friable and thus readily dirtied the fur of the mice. This explains why, in the high fat diet groups, the coat state score was generally higher than in the regular diet groups.

In the regular diet group, the Kruskall-Wallis test indicates a significant difference between groups at the end of the first and the second UCMS procedure (week 7: H_3, 33_ = 25.1, *p*<0.001; week 20: H_3, 33_ = 20.2, *p*<0.001). The UCMS regimen induced a deterioration of the coat state (week 7: U = 0.0, *p*<.001; week 20: U = 1.5, *p*<.001). Importantly, fluoxetine treatment significantly reversed the deterioration of the coat state induced by UCMS (week 7: U = 8, *p*<.05; week 20: U = 6.5, *p*<.01).

Similarly, in the high fat diet group, the Kruskall-Wallis test indicates a significant difference between groups at the end of both the first and the second UCMS regimen (week 7: H_3, 38_ = 8.5, *p*<.05; week 20: H_3, 38_ = 15.9, *p*<0.01). However, the UCMS-induced coat state degradation (week 7: U = 16, *p*<.01; week 20: U = 10.5, *p*<.01) was not significantly reversed by chronic fluoxetine (week 7: U = 32, *ns*; week 20: U = 46.5; *ns*).

An overall gain in body weigh with time was observed in each group (Friedman test: Control/NaCl: RD: X^2^_9, 20_ = 114.9, *p*<.001; HFD: X^2^_10, 20_ = 38.8, *p*<.01; Control/fluoxetine: RD: X^2^_9, 20_ = 159.6, *p*<.001; HFD: X^2^_7, 20_ = 59.7, *p*<.001; UCMS/NaCl: RD: X^2^_8, 20_ = 127.1, *p*<.001; HFD: X^2^_10, 20_ = 70.1, *p*<.001; UCMS/fluoxetine: RD: X^2^_10, 20_ = 89.2, *p*<.01; HFD: X^2^_10, 20_ = 117.3, *p*<.001). The Kruskall-Wallis test failed to establish any difference between groups at the end of the first UCMS procedure (week 7: H_7, 71_ = 11.8, *ns*), and at the end of the second UCMS procedure (week 20: H _7, 71_ = 12.6, *ns*) (data not shown).

### Splash test

The Friedman test indicates a significant difference over the four sessions of the splash test for each group (Control/NaCl: RD: X^2^_9, 3_ = 16.9, *p*<.001; HFD: X^2^_10, 3_ = 20.5, *p*<.001; Control/fluoxetine: RD: X^2^_10, 3_ = 17.5, *p*<.001; HFD: X^2^_8, 3_ = 15.9, *p*<.01; UCMS/NaCl: RD: X^2^_8, 3_ = 7.5, *p* = .05; HFD: X^2^_10, 3_ = 9.5, *p*<.05; UCMS/fluoxetine: RD: X^2^_6, 3_ = 12.6, *p*<.01; HFD: X^2^_10, 3_ = 16.7, *p*<.001).

**Figure 3:**
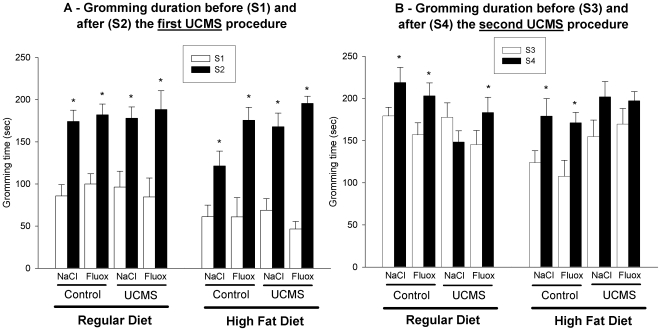
Total grooming time in the splash test as an index of motivational and self-care behaviour. The grooming time (Mean +/− SEM) is presented for the control and the unpredictable chronic mild stress (UCMS) groups treated or not with fluoxetine (10 mg/kg, administrated daily during the last 5 weeks of each UCMS procedure) in the regular and high fat diet conditions. A) Before (S1) and after (S2) the first UCMS procedure. *p<.05: comparison between S1 and S2 for each group. B) Before (S3) and after (S4) the second UCMS procedure. *p<.05: comparison between S3 and S4 for each group.

During the first UCMS procedure, the total grooming time increased between the beginning (Session S1) and the end (Session S2) of the regimen in each group suggesting an increase in the motivation for grooming and the fact that the mice became used to the sucrose solution (Figure 3A, Control/NaCl: RD: T = 2, *p*<.01; HFD: T = 2, *p*<.01; Control/fluoxetine: RD: T = 7, *p*<.05; HFD: T = 1, *p*<.01; UCMS/NaCl: RD: T = 0.0, *p*<.05; HFD: T = 0.0, *p*<.01; UCMS/fluoxetine: RD: T = 0.0, p<.05; HFD: T = 0.0, p<.01).

During the second UCMS procedure, we also found an increase in the total time of grooming behaviour in control mice who received a regular or a high fat diet (Figure 3B, Control/NaCl: RD: T = 5, *p*<.05; HFD: T = 0.0, *p*<.01; Control/fluoxetine: RD: T = 0.0, *p*<.01; HFD: T = 1, *p*<.01) while UCMS abolished this increase in grooming motivation (RD: T = 5, *ns*; HFD: T = 12, *ns*). Fluoxetine restored the total grooming time to the unstressed level in UCMS mice who received a regular diet, but this was not observed in the mice with the high fat diet (RD: T = 0.0, *p*<.05; HFD: T = 24, *ns*) suggesting that a high fat diet prevents the reversal effect of fluoxetine on repeated UCMS-induced grooming disturbance.

### Reward maze test

Regarding the latency to pass the first door and the number of passage through the second door, the Kruskall-Wallis tests failed to establish any differences between groups both at the end of the first and at the end of the second UCMS procedure (Latency to pass the first door: S1: H_7, 71_ = 8.4, *ns*; S2: H_7, 71_ = 5.6, *ns*. Number of passage: S1: H_7, 71_ = 8.7, *ns*; S2: H_7, 71_ = 13.3, *ns*). These results demonstrate that both high fat diet, UCMS and fluoxetine does not induce an anxiety-like state and perturbation of the locomotor and exploratory behaviour (Data not shown).

**Figure 4:**
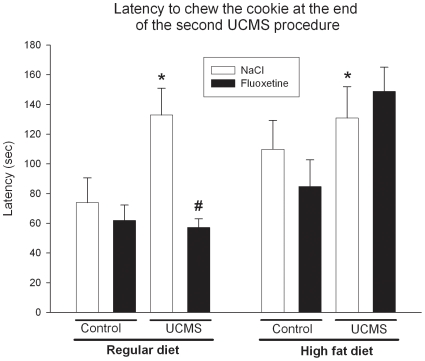
Latency to chew a cookie in the reward maze test as an index of anhedonia. The latency to chew the chocolate cookie (Mean +/− SEM) is given for the session performed at the end of the second unpredictable chronic mild stress (UCMS) procedure (R2). The results are presented for the Control and UCMS mice under a regular or a high fat diet regimen who receive during the last 5 weeks of each UCMS procedure daily injection of either NaCl (9%) or Fluoxetine (10 mg/kg). *p<.05: Comparison to the control/NaCl group in the regular diet condition. #p<.05: Comparison between UCMS/NaCl and UCMS/fluoxetine groups in the same diet condition.

Regarding the latency for chewing the chocolate cookie at the end of the first UCMS regimen, the Kruskall-Wallis test indicates no significant difference between the groups (S1: H_7, 71_ = 9.3, *ns*). However, during the second UCMS procedure, the latency in chewing the chocolate cookie was significantly different between groups at the end of the regimen (Figure 4, S2: H_7, 71_ = 19.4, *p*<.01). In the control groups, nor high fat diet and fluoxetine treatment induced a modification of the latency to chew the cookie (HFD vs RD: Control, NaCl: U = 26, *ns* Control, fluoxetine: U = 29, *ns*. NaCl vs fluoxetine: Control, RD: U = 41, *ns*; Control HFD: U = 31.5, *ns*) indicating that no anhedonic effect was induced by the high fat diet. The UCMS significantly increased the latency to chew the reward in both the regular and the high fat diet conditions (RD: U = 12, *p*<.05; HFD: U = 19, *p*<.05). Fluoxetine only counteracted this effect in mice who received a regular diet (RD: U = 5, *p*<.05; HFD: U = 45, *ns*). These results indicate that high fat diet prevents the reversal effect of fluoxetine on UCMS-induced anhedonia.

## Discussion

Based on the hypothesis that cardiovascular risk factors induce poor response to AD treatment in depressed subjects, the main objective of the present study was to develop a naturalistic animal model of AD resistance associating UCMS, a valid animal model of depression, with cardiovascular risk factors, especially through a high fat diet regimen. A high fat diet regimen inducing hypercholesterolemia is considered as a risk factor for the development of atherosclerosis. However in mice, the susceptibility to high fat diet is strain dependant. The BALB/c mice that are the most sensitive to the UCMS procedure need a longer period of high fat diet to react of the cardiovascular effects of this diet [32]. To ensure the effectiveness of the high fat diet and since resistance to AD is increased with recurrent depressive episodes, we designed a two UCMS procedure in BALB/c mice to mimic recurrent depression in humans. Our results clearly demonstrate that UCMS-induced behavioural changes including coat state degradation, disturbances in grooming motivation (splash test) and decrease in the motivation to obtain a palatable stimulus (reward maze test), were all reversed by fluoxetine treatment in mice subjected to a regular diet. However, in all these behavioural tests, a high fat diet regimen abolished the ability of the AD fluoxetine to reverse UCMS-induced depressive-like state at the end of the second period of the UCMS procedure.

### Does fluoxetine reverse UCMS-induced depression-like behaviour?

In the regular diet condition, in spite of difference in the time of occurrence, the behavioural alterations induced by UCMS observed in this study replicate the behavioural changes reported in previous studies using the UCMS protocol in mice [26], [31], [33], [34], [35]. Ordinarily, a single UCMS regimen is sufficient to induce behavioural alterations, which was replicated here on the coat state. However, the UCMS-effects in the splash test and in the reward maze test were observed only after the second challenge. The late-onset apparition of the UCMS effect can be explained by the number, the intensity and the frequency of the different stressors application: indeed, as mice were exposed to a second UCMS, we reduced the severity of the stressors, mainly for ethical reasons.

UCMS and fluoxetine produced their usual effect on the coat state test, since the UCMS-induced coat state degradation was prevented by fluoxetine during both the first and the second UCMS procedure. On the body weight, the absence of UCMS and fluoxetine effect is in accordance to other studies using fluoxetine treatment in the UCMS model of depression [34], [39]. The results obtained in the splash test suit those of the coat state since UCMS-induced disturbance in grooming behaviour was reversed by fluoxetine treatment. Indicating a loss of motivational and self care behaviour in mice subjected to two UCMS procedure, the disturbance in grooming behaviour is considered to parallel the motivational and apathetic behaviour observed in depression [36]. The reward maze test takes advantage of multiple measures to give a more accurate analysis of the UCMS-induced effect on the behaviour, and particularly on anhedonia, which represents a core symptom of human depression. Our results show that UCMS procedure does not induce an anxiety-like state and perturbations of locomotor and exploratory behaviour. However, UCMS induced anhedonia, an effect that was counteracted by fluoxetine treatment. Taken together, these results suggest through multiple behavioral readouts that chronic AD treatment is effective in reversing a depression-like phenotype induced by UCMS.

In our study, the behavioural alterations induced by two UCMS procedures results in symptoms paralleling human depression and the pharmacological reversal with chronic fluoxetine models the successful treatment of recurrent depression. Furthermore, this model provides a powerful tool to describe a model of “treatment-resistant depression” based on the hypothesis that cardiovascular risk factors-induced AD resistance.

### Does the high fat diet regimen induce depression resistance to fluoxetine treatment?

In the high fat diet conditions, UCMS induced behavioural alterations similar to those observed in the regular diet condition. However, the high fat diet regimen prevented chronic fluoxetine treatment from reversing UCMS-induced depression-like behaviour. This result cannot be explained by the cumulative period of UCMS and the previous treatment and withdrawal to fluoxetine since the fluoxetine resistance was not observed in the regular diet condition.

In the coat state test, fluoxetine was unable to improve UCMS-induced coat state degradation during the first and the second UCMS procedure in mice fed a high fat diet. However, the deterioration of the coat state induced by the crisp food texture itself makes this variable difficult to interpret as the observed-effect might be unspecific and unrelated to depressive-like state. In the splash test, which also represents an index of grooming behaviour, results clearly indicate that the UCMS-induced grooming disturbance was not reversed by fluoxetine treatment at the end of the second procedure. In this test, where the effect of the food texture was excluded as an explanation to the observed effects, only the high fat diet regimen can explain the fluoxetine resistance in reversing motivational and self-care behaviour. Finally, in the reward maze test, we found that high fat diet *per se* has no anhedonic effect in spite of an increase chewing latency in the control mice. This effect can be explained by the difference in palatability between the regular and the high fat diet which could interfere with the behavioural changes observed. Nevertheless, the UCMS-induced increase of the chewing latency for the cookie in both conditions can be interpreted as UCMS-induced anhedonic behaviour. Similar to the regular diet conditions, UCMS-induced anhedonia in the high fat diet condition, however, in contrast, fluoxetine was unable to reverse these effects of UCMS in mice who received a high fat diet regimen.

In the high fat diet conditions, through multiple behavioural paradigms, we demonstrated that a high fat diet prevented chronic fluoxetine treatment from reversing UCMS-induced depression-like phenotype. These results are important because they mimic AD resistance induced by vascular risk factors. The hypothesis of AD resistance induced by vascular risk factors is based on a number of clinical studies which demonstrate the same effect on patients with vascular risk factors or suffering from cardiovascular disease. Several studies suggest that depressed patients with cerebrovascular lesions show increased resistance to both AD treatment and electroconvulsive therapy [5], [6], [40], [41], [42]. AD resistance is also associated with the presence of vascular risks factors. A high incidence of depression has been reported when associated with the total burden of vascular risk factors [43] and this so-called cardiovascular risk score (sum of vascular risk factors including aging, smoking cigarette, history family of premature vascular disease, hypertension, diabetes and hypercholesterolemia) is associated with low rates of remission [9]. Indeed, poor long-term outcomes of depressive symptoms and low rates of remission with selective serotonin reuptake inhibitor (SSRI) treatment are associated with cumulated cardiovascular risk factors and can be predicted by the burden of cardiac disease [8], [44].

By mimicking clinical data our results consequently allow us to suggest that the combination of UCMS with a high fat diet can be used as an animal model of resistance to AD drugs. However, in order to definitely confirm that we modelled AD resistance induced by vascular risk factors, future studies should use different tests that are not based on food reward such as the resident/intruder test, the tail suspension test or the forced swim test which have already been used to assess UCMS effects [22], [35], [45]. It is also very important to generalise our results both with other AD drugs including other SSRIs but also other classes of AD and with other cardiovascular risk factors and finally, to check whether these results are not related to a shift in the dose-response to fluoxetine using higher dose of AD.

Clinical data demonstrate the need for animal models in order to study AD resistance. We suggest that a naturalistic animal model of AD resistance related to sub-nosographic clinical entities, associating UCMS with cardiovascular risk factors, especially a high fat diet regimen, could prove to be extremely useful. This original model could serve as useful tool for future research and eventually lead to improvements in the treatment of depression and possibly to increased survival in patients with cardiovascular disease by aggressively treating the cardiovascular risk factors, or by selectively treating depression with drugs that also modify these risk factors. Future research in this field could open up new avenues for the development of novel AD or other treatment strategies for these patients.
